# SIRT1, resveratrol and aging

**DOI:** 10.3389/fgene.2024.1393181

**Published:** 2024-05-09

**Authors:** Blanka Rogina, Heidi A. Tissenbaum

**Affiliations:** ^1^ Department of Genetics and Genome Sciences, School of Medicine, University of Connecticut Health Center, Farmington, CT, United States; ^2^ Institute for Systems Genomics, Farmington, CT, United States; ^3^ Department of Molecular, Cell and Cancer Biology UMass Chan Medical School, Worcester, MA, United States

**Keywords:** aging, sirtuins, SIRT1, resveratrol, calorie restriction, dietary supplements

## Abstract

Aging is linked to a time-associated decline in both cellular function and repair capacity leading to malfunction on an organismal level, increased frailty, higher incidence of diseases, and death. As the population grows older, there is a need to reveal mechanisms associated with aging that could spearhead treatments to postpone the onset of age-associated decline, extend both healthspan and lifespan. One possibility is targeting the sirtuin SIRT1, the founding member of the sirtuin family, a highly conserved family of histone deacetylases that have been linked to metabolism, stress response, protein synthesis, genomic instability, neurodegeneration, DNA damage repair, and inflammation. Importantly, sirtuins have also been implicated to promote health and lifespan extension, while their dysregulation has been linked to cancer, neurological processes, and heart disorders. SIRT1 is one of seven members of sirtuin family; each requiring nicotinamide adenine dinucleotide (NAD^+^) as co-substrate for their catalytic activity. Overexpression of yeast, worm, fly, and mice SIRT1 homologs extend lifespan in each animal, respectively. Moreover, lifespan extension due to calorie restriction are associated with increased sirtuin activity. These findings led to the search for a calorie restriction mimetic, which revealed the compound resveratrol; (3, 5, 4′-trihydroxy-trans-stilbene) belonging to the stilbenoids group of polyphenols. Following this finding, resveratrol and other sirtuin-activating compounds have been extensively studied for their ability to affect health and lifespan in a variety of species, including humans via clinical studies.

## 1 Introduction

Aging is associated with a progressive metabolic, physiological decline and can be genetically and environmentally modified (Helfand and Rogina, 2000). The search for the molecular basis of aging led to the identification of several pathways associated with longevity including insulin/IGF-1, target of rapamycin (TOR) and the Sirtuins (Kenyon, 2010; Chen et al., 2022). The sirtuins are a family of nicotinamide adenine dinucleotide (NAD^+^)-dependent histone deacetylases (Haigis and Sinclair, 2010; Hall et al., 2013; Bonkowski and Sinclair, 2016; Dai et al., 2018; Singh et al., 2018). Sirtuins are also categorized as deacetylases because they catalyze the post-translational modification of signaling molecules including decrotonylation, ADP-ribosylation, diacylation, desuccinylation, demalonylation, depropynylation, delipoamidation, and deglutarylation, and other long-chain fatty acid deacylations (Feldman, Baeza, and Denu, 2013; Choudhary et al., 2014; Fiorentino et al., 2022).

In mammals, there are seven members (SIRT1-SIRT7) including SIRT1, SIRT6 and SIRT7, which are localized to the nucleus, and SIRT3, SIRT4, and SIRT5 localized to the mitochondria, SIRT2 localized to the cytosol, and SIRT1 also localized to cytosol in some cell types (Bonkowski and Sinclair, 2016). As histone deacetylases, sirtuins function by removing acetyl groups from the target proteins resulting in either inhibition or activation. SIRT1, SIRT6 and SIRT7 have many functions including: regulators of transcription, control of cellular metabolism, DNA repair, cell survival, tissue regeneration, inflammation, circadian rhythms and neuronal signaling (Haigis and Sinclair, 2010). SIRT3-5 are important for switching to mitochondrial oxidative metabolism during CR and modulate stress tolerance (Verdin et al., 2010).

SIRT1 as predominantly a nuclear protein, deacetylates histones H3, H4 and H1, however, it also modifies more than 50 non-histone proteins including transcriptional factors p53, peroxisome proliferator-activated receptor-γ co-activator 1α (PGC1α), p65, NF-κB, sterol regulatory element-binding protein (SREBP), and repair proteins (Ku-70, PARP1), and others (Haigis and Sinclair, 2010; Bonkowski and Sinclair, 2016).

In general, sirtuins are unique and require nicotinamide adenine dinucleotide (NAD^+^) as a cofactor for their enzymatic activities. Aging is characterized with decline in NAD^+^ availability, and reduced sirtuin activities leading to possibility that increased sirtuin activity may ameliorate the negative effects of age-related decline (Imai and Guarente, 2014; Imai and Guarente, 2016). The original connection between longevity and the sirtuins began with studies in the budding yeast *Saccharomyces cerevisiae*. The budding yeast ortholog to SIRT1, Sir2 (silent information regulator 2) was found to increase yeast mother cell replicative lifespan when yeast are provided with additional SIR2 copies (Kaeberlein et al., 1999). Further studies in model organisms showed that similarly, SIRT1 homologs in worms, flies, or mice lead to improved health and increased longevity when SIRT1 activity is increased (Kaeberlein et al., 1999; Tissenbaum and Guarente, 2001; Rogina and Helfand, 2004; Baur et al., 2010; Satoh et al., 2013).

A second connection of SIRT1 to longevity was a coupling to calorie restriction (CR), where caloric intake is reduced but without malnutrition, and CR can extend lifespan and health across phylogeny (Al-Regaiey, 2016; Green et al., 2022). In response to CR, there is increased SIRT1 activity (Cantó and Auwerx, 2009). SIRT1 is also associated with CR associated lifespan extension as deletion of SIRT1 prevents longevity extension by CR in budding yeast, worms, and flies. Therefore, these data suggest that SIRT1 most likely mediates longevity extension in CR (Lin et al., 2000; Rogina and Helfand, 2004; Wang and Tissenbaum, 2006). In addition, mammalian SIRT1, SIRT3 and SIRT6 are all linked to CR, their expression is induced by CR and exercise, while their genetic ablation prevents benefits of both CR and exercise (Chen et al., 2005; Dai et al., 2018). In addition, over-expression of SIRT1 or SIRT6 in mice mimic physiological effects observed in CR and exercise, and extends lifespan (Kanfi et al., 2012; Satoh et al., 2013).

Identification of SIRT1 upregulation following CR and its importance in mediating the benefits of CR prompted a search for CR mimetics, an endeavor that led to identification of several polyphenolic compounds. Resveratrol (discussed in greater detail below) was identified as the strongest yeast sirtuin activator (Howitz et al., 2003) and has been shown to improve health and survival of flies and mice on a high-calorie diet (Wood et al., 2004a; Wood et al., 2004b; Baur et al., 2006). In mice, resveratrol improves adipose insulin signaling; and in rhesus monkeys on a high-fat diet, resveratrol reduces inflammation in adipose tissue (Baur et al., 2006; Jimenez-Gomez et al., 2013; Dai et al., 2018). Therefore, SIRT1 has been attractive as a potential pharmacological target for anti-aging and preventing age-related diseases in humans, resulting in the initiation of clinical studies (Dai et al., 2018). Together, these findings in non-human model organisms and the conserved sirtuin function across species support the notion that SIRT1 is a promising target to mediate impacts of aging.

## 2 SIRT1 and lifespan

The role of Sirtuins in the aging process was first linked to lifespan when it was shown that adding an extra copy of yeast SIRT1, Sir2, increased yeast mother cell replicative lifespan (Kaeberlein et al., 1999). In budding yeast, longevity extension by sir2 is linked to CR, a finding supported by studies showing that CR does not extend lifespan of yeast sir2 mutant (Lin et al., 2000). Increased dosage of SIRT1 homolog in worms obtained by using a *Caenorhabditis elegans* strain that contains duplication *sir-2.1* (*C. elegans* Sirt1) extends worm longevity (Tissenbaum and Guarente, 2001; Viswanathan and Tissenbaum, 2013). This longevity extension is associated with CR (Wang and Tissenbaum, 2006). In flies, increased expression of Sirt1 (formerly Sir2) either ubiquitously, in neurons, or in the adult fat body extends lifespan (Rogina and Helfand, 2004; Bauer et al., 2008; Frankel, Ziafazeli, and Rogina, 2011; Banerjee et al., 2012; Whitaker et al., 2013). This *Drosophila* longevity extension is dose-dependent, with extension observed only when Sirt1 levels are increased two and five fold (Whitaker et al., 2013) but not when the levels are overexpressed at high levels which can induce cellular toxicity via increased expression of the JNK-signaling pathway (Burnett et al., 2011; Whitaker et al., 2013). Experiments in flies have also provided additional evidence linking longevity in Sirt1 overexpressing flies to a mechanism akin to CR. Longevity extension in CR flies was blocked by mutations in Sirt1, and CR increases Sirt1 levels, confirming that Sirt1 is required for CR longevity extension (Rogina et al., 2002; Rogina and Helfand, 2004; Banerjee et al., 2012). In the same studies, CR did not further extend the lifespan of long-lived Sirt1 overexpressing flies (Rogina and Helfand, 2004; Banerjee et al., 2012). In a different study in CR flies, Sirt1 mRNA was increased (Rogina et al., 2002), suggesting at least partial overlap between the Sirt1 and CR longevity pathways. Beyond increasing longevity, increased activity of Sirt1 mediates enhanced spontaneous physical activity associated with CR in the fly (Parashar and Rogina, 2009; 2010; Woods et al., 2014). In mammalian studies, transgenic mice have provided substantial insight into sirtuin function and the overlap of Sirt1 with CR, including requirements of SIRT1 for increased activity (Chen et al., 2005). SIRT1 transgenic mice overexpressing high levels of SIRT1 display a phenotype similar of CR, including reduced blood lipid levels and improved glucose metabolism but did not experience increased lifespan (Bordone et al., 2007). Notably, CR also increases SIRT1 activity in mice, further supporting the notion that SIRT1 and CR longevity pathways are overlapping (Chen et al., 2005). Therefore, across many studies in model systems, SIRT1 function overlaps with CR.

SIRT1 transgenic mice have also revealed other connections to the aging process. Whole-body SIRT1 moderate overexpression improved healthy aging and protected mice from metabolic-syndrome-associated cancer, preventing metabolic damage induced by a high-fat diet; however, this SIRT1 overexpression failed to extend longevity. While, brain-specific overexpression of SIRT1 is sufficient to increase lifespan in both males and females mice (Satoh et al., 2013). These mice have increasing neural activity through elevated expression of the orexin type 2 receptor (*Ox2r*), stimulating the sympathetic nervous system and maintaining muscle mitochondrial morphology and function, physical activity and oxygen consumption during aging (Satoh et al., 2013). These findings suggest that SIRT1-overexpression in the brain plays a key role in regulating aging and longevity in mammals, and is consistent with fly studies in which neuronal Sirt1 overexpression is sufficient to increase fly lifespan (Rogina and Helfand, 2004; Pfluger et al., 2008; Herranz et al., 2010; Satoh et al., 2013). There are additional studies that link SIRT1 and brain function and age-associated neurodegenerative diseases. Overexpression of SIRT1 reduces amyloid plaques in several models of Alzheimer’s diseases by inhibiting NF-κB signaling (Donmez and Outeiro, 2013). However, some studies show that inhibition of SIRT1 protects neurons, illustrating a complex role of SIRT1 in both pro- and anti-aging in mice (Li et al., 2008). Taken together, since its original studies in yeast, SIRT1 has emerged as a major regulator of longevity and health. Although most research has been completed in non-human model organisms, increased SIRT1 levels were found in humans participating in CR, suggesting a universal role of sirtuins and its effects of CR (Civitarese et al., 2007).

## 3 Resveratrol: history, potential and challenges

Resveratrol was identified in 2003 during a high-throughput screen of small molecules that function as allosteric activators of yeast SIRT1 in an attempt to identify a CR mimetic (Howitz et al., 2003). Compounds identified during this screening included stilbenes (such as resveratrol), chalcones (such as butein), and flavone (for example, quercetin) (Howitz et al., 2003) These compounds lowered the binding affinity of SIRT1 for the substrate leading to increase in enzymatic activity. In this original study, resveratrol, a component of red wine, was the most potent SIRT1 activator and mimicked CR in yeast. Resveratrol was reported to stimulate SIRT1, increase DNA stability and extend yeast lifespan by 70% (Howitz et al., 2003). Studies in worms and fruit flies confirm that resveratrol and fisetin, another sirtuin activator, activate sirtuin’s activity and extend lifespan without affecting reproduction (Wood et al., 2004a). Since reproduction in female flies is associated with a cost of mating, egg production and egg laying negatively affect fly lifespan (Rogina et al., 2007), it is important to note that longevity associated with resveratrol, was not due to decreased female reproduction. Importantly, longevity was also not extended when Sirt1 mutant either worms or flies were subject to CR and given resveratrol, confirming that resveratrol extends longevity through a mechanism that mimics CR (Wood et al., 2004a; Rogina and Helfand, 2004).

In middle-aged mice on a high-calorie diet, resveratrol was shown to improve health and survival (Baur et al., 2006). These mice had increased insulin sensitivity, reduced insulin-like growth factors, increased mitochondrial number, and improved motor functions, among other benefits (Baur et al., 2006). In non-human primates, resveratrol suppresses body mass gain in a seasonal model of obesity without affecting spontaneous locomotor activity (Dal-Pan, Blanc, and Aujard, 2010). Further, resveratrol reverses age-associated proinflammatory phenotypes in non-human primate vascular smooth muscle cells, prevents age-related DNA and RNA oxidative damage, and selectively enhances cognitive performances (Dal-Pan et al., 2011; Csiszar et al., 2012; Marchal et al., 2013). A recent study showed that resveratrol reduces mitochondrial loss and promotes autophagy in skeletal muscle of non-human primates following a long-term high fat/sugar diet (Hyatt et al., 2023).

Almost 200 clinical studies, over the last 20 years, have evaluated the safety and effects of resveratrol in humans (Brown et al., 2024). Studies have included healthy individuals to study metabolism, safety, pharmacokinetics, and the bioavailability, as well to study health maintenance, and a disease prevention. Resveratrol has also been evaluated in variety of clinical indications including patients with different metabolic, cardio-metabolic disorders, cardiovascular diseases, T1 Diabetes, T2 Diabetes, Non-Alcoholic Fatty Liver Disease (NAFLD), coronary heart disease, hypercholesterolemia, patients with atherosclerosis, obese healthy individuals, and overweight individuals with mildly elevated blood pressure (Brown et al., 2024). These studies revealed that resveratrol can improve glucose metabolism and cardiovascular disease markers. Other studies have included brain ischemic stroke patients, women with pregnancy-induced eclampsia, women with endometriosis, children with autism spectrum disorder, patients with cancer, mental health among others (reviewed in Brown et al., 2024). These studies revealed effects of resveratrol as improving glucose metabolism and cardiovascular disease markers, as well as in reducing oxidative stress, inflammation, and cell death. Resveratrol can have neuroprotective, antidiabetic, antibacterial and anti-aging effects and delays cognitive decline in Alzheimer’s disease patients (Turner et al., 2015; Brown et al., 2024). Clinical trials have revealed promising results in wide range of applications including T2 diabetes, metabolic syndrome, NAFLD. Currently, there are about two dozen ongoing clinical trials for resveratrol with some enrolling participants as this review is being written.

However, despite its potency in non-human models, suggestive results and human clinical studies, resveratrol has low bioavailability in humans and thus low clinical potency. It is important to note that the effects of resveratrol are dose dependent. Studies in flies show that maximal longevity effect was observed at 200 μmol in females and at 100 μmol in males (Wood et al., 2004a). In yeast, low doses of resveratrol increase yeast lifespan by 70% but higher doses have only minor effects (Howitz et al., 2003). In addition to affecting SIRT1, resveratrol, interacts with many other proteins. Resveratrol targets include AMPK, complex III of the mitochondrial electron transport chain, PARP1, phosphodiesterase, among others (Bonkowski and Sinclair, 2016). To avoid these off-target impacts, several other less potent SIRT1 activators have been identified, such as piceatannol, which has been used in many preclinical trials and shows potential to prevent or impede growth of different cancers (Banik et al., 2020). Synthetic Sirtuin-activating compounds (STAC) such as SRT1720 and SRT2104, compounds unrelated to resveratrol, have been identified as potent SIRT1 activators. It was shown that these STACs extend mouse healthspan and lifespan (Minor et al., 2011; Mercken et al., 2014). SRT1720 improves both mean and maximum lifespan and healthspan in obese mice, including reduced liver steatosis, increased insulin sensitivity, enhanced locomotor activity without any observed toxicity (Minor et al., 2011). These compounds have been also used in preclinical and clinical studies (Hoffmann et al., 2013; Krueger et al., 2015).

## 4 SIRT1 inhibitors

SIRT1 directly or indirectly affects many histone and non-histone targets and has been implicated in variety of physiological processes including gene regulation, apoptosis, senescence, autophagy, proliferation, genome stability, tumorigenesis and aging, which are beneficial for an organism (Haigis and Sinclair, 2010). SIRT1 has key roles in the DNA damage response, as a deacetylase of protein involved in DNA repair. SIRT1 protects against UV-induced human fibroblast damage and decreases levels of oxidative stress biomarker in the hippocampus of rats treated with ketogenic diet (Elamin et al., 2018). SIRT1 targets include the tumor suppressor gene p53, which regulates repair of DNA damage, apoptosis, the cell cycle proteins and proliferation (Vaziri et al., 2001). SIRT1 deacetylation of p53 inhibits transcriptional activity of p53. However, SIRT1 has also been implicated in disorders and diseases. SIRT1 is both a tumor suppressor and a tumor promoter, which depends on cell type and SIRT1 localization (Alves-Fernandes and Jasiulionis, 2019). SIRT1 upregulation is associated in acute myeloid leukemia (AML), and primary, prostate, colon, melanoma and non-melanoma skin cancers. On the other hand, SIRT1 downregulation was described in breast cancer and hepatic cell carcinomas (Alves-Fernandes and Jasiulionis, 2019). SIRT1 deacetylation of mutant huntingtin prevents its degradation, thus inhibition of SIRT1 could be beneficial for patients suffering from Huntington’s Disease (HD) (Dai et al., 2018; Fiorentino et al., 2022). Hence, Selisistat (EX-527), a SIRT1 selective inhibitor, is used in the clinic for patients with HD (Süssmuth et al., 2015).

## 5 Discussion

**FIGURE 1:**
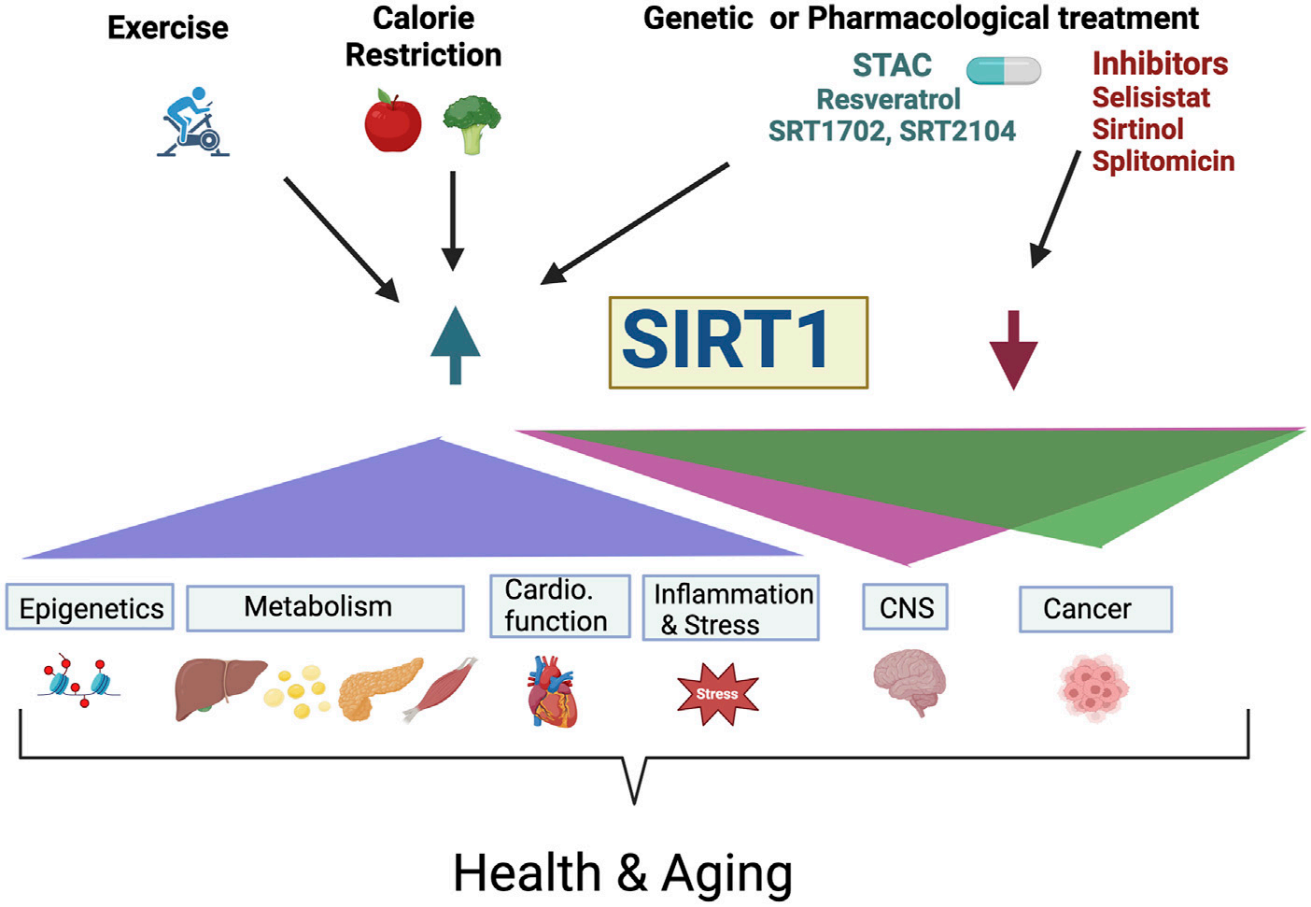
The role of SIRT1 in health and aging. Exercise, calorie restriction, genetic and pharmacological activation or inhibition of SIRT1 activity modulate physiological processes including epigenetic, metabolism, cardiovascular function, inflammation, stress, central nervous system function and cancer resulting to promote healthspan and lifespan. Pharmacological activation of SIRT1 levels/activity (Resveratrol, SRT1702, and SRT2104) or inhibition (Selisistat, Sirtinol, Splitomicin, and others), have the potential to be used for treatment of neurodegenerative disease and cancer. For example, the SIRT1 agonist, resveratrol improves memory deficit in Alzheimer’s disease and has protective effects on amyotrophic lateral sclerosis (ALS), but SIRT1 inhibitions such as Selisistat (EX-527), is used in the clinic for patients with Huntington Disease. STAC, Sirtuin-activating compounds; Cardio. Function, Cardiovascular Function; CNS, Central Nervous System.

SIRT1 is a key epigenetic regulator that affects transcriptional profiles and genome stability of many regulatory pathways. It has diverse effects on many aspects of physiology including metabolism, cell survival, stress response, inflammation, circadian rhythm, replicative senescence, neurodegeneration, and cancer, among others, (Figure 1). SIRT1 protects against DNA damage, and modulates DNA repair. There is a growing support for the role of SIRT1 in mediating longevity in a variety of organisms and has been implicated as a major mediator of health benefits associated with CR, (Figure 1). There is also continuing search for identification of STACs, such as resveratrol, that could be used to alleviate or postpone age-related diseases and promote healthy aging. There has been much discussion on the effects of SIRT1 and other sirtuins and many labs have contributed to improve our understanding of its biological role and its enzymatic activity (Bonkowski and Sinclair, 2016; Dai et al., 2018; M; Chen et al., 2024). This remarkable body of work provides a framework and promise for future studies on the role of SIRT1, and its activators in health, disease and aging.
